# Canadian report card on health equity across the life-course: Analysis of time trends and cross-national comparisons with the United Kingdom

**DOI:** 10.1016/j.ssmph.2018.09.009

**Published:** 2018-09-25

**Authors:** Alexandra Blair, Arjumand Siddiqi, John Frank

**Affiliations:** a Département de Médecine Sociale et Préventive, École de Santé Publique, Université de Montréal, 7101 Park Ave, Montréal, Québec, Canada H3N 1X9; b Centre de Recherche du Centre hospitalier de l’Université de Montréal (CRCHUM), 850 St Denis St, Montréal, Québec, Canada H2X 0A9; c Scottish Collaboration for Public Health Research and Policy, University of Edinburgh, 20 West Richmond Street, Edinburgh EH8 9DX, United Kingdom; d Division of Epidemiology, Dalla Lana School of Public Health, University of Toronto, 155 College Street, Toronto, Ontario, Canada M5T 3M7; e Department of Health Behavior, Gillings School of Global Public Health, University of North Carolina at Chapel Hill, 302 Rosenau Hall, CB #7440, Chapel Hill, NC 27599-7440, United States; f Usher Institute of Population Health Sciences and Informatics, University of Edinburgh, Old Medical School, Teviot Place, Edinburgh EH8 9AG, United Kingdom

**Keywords:** ECEC, Early childhood education and child care, CANSIM, Canadian Socio-Economic Information Management System, CCS, Canadian Cancer Society, CIHI, Canadian Institute for Health Information, GDP, Gross domestic product, OECD, Organisation for Economic Co-operation and Development, ONS, Office for National Statistics, SDoH, Social determinants of health, UK, United Kingdom, WHO, World Health Organization, Canada, United Kingdom, Health equity, Health and social policy, Lifecourse epidemiology, Public health

## Abstract

Addressing social determinants of health (SDoH) has been acknowledged as an essential objective for the promotion of both population health and health equity. Extant literature has identified seven potential areas of investment to address SDoH: investments in sexual and reproductive health and family planning, early learning and child care, education, universal health care, as well as investments to reduce child poverty, ensure sustainable economic development, and control health hazards. The aim of this paper is to produce a ‘report card’ on Canada’s success in reducing socioeconomic and health inequities pertaining to these seven policy domains, and to assess how Canadian trends compare to those in the United Kingdom (UK), a country with a similar health and welfare system. Summarising evidence from published studies and national statistics, we found that Canada’s best successes were in reducing socioeconomic inequalities in early learning and child care and reproductive health—specifically in improving equity in maternal employment and infant mortality. Comparative data suggest that Canada’s outcomes in the latter areas were like those in the UK. In contrast, Canada’s least promising equity outcomes were in relation to health hazard control (specifically, tobacco) and child poverty. Though Canada and the UK observed similar inequities in smoking, Canada’s slow upward trend in child poverty prevalence is distinct from the UK’s small but steady reduction of child poverty. This divergence from the UK’s trends indicates that alternative investment types and levels may be needed in Canada to achieve similar outcomes to those in the UK.

## Highlights

• We summarize trends in health equity in Canada and compare Canadian trends to those in the United Kingdom (UK).
• Trends were assessed according to seven previously-identified domains of societal-level investments.
• Canada’s best improvements were in early learning and care and reproductive health. Trends were similar to those in the UK.
• Canadian equity trends in employment and universal health care were stable, and outperformed by those in the UK.
• Canada’s least promising trends were in smoking and child poverty. Unlike in the UK, child poverty has increaesd in Canada.

## 1 Introduction

Socioeconomic inequalities in health are known to result from societal socioeconomic inequalities—experienced even before birth and accumulated throughout life (Marmot et al., 2010). With the aim of improving both health and well-being for all, and reducing health inequities, extant reports on the Social Determinants of Health (Marmot et al., 2008, Marmot et al., 2010; Wilkinson & Marmot, 2003) have identified several areas of investment (Frank et al., 2015). These can be summarized into seven domains: 1) sexual and reproductive health, family planning, and pre- and perinatal care, 2) labour market and tax policies to reduce child poverty, 3) early childhood education and care, 4) secondary and post-secondary education, 5) accessible and high-quality primary, secondary, and tertiary health care, 6) economic and marketing controls on health hazards, and 7) sustainable economic development to support meaningful employment. Though many of these areas overlap, and alternative classification systems can be used, this broad taxonomic classification of investment areas offers a valuable framework to guide the study and interpretation of health equity-related outcomes. These investment areas were identified as priorities for their ability to shift distributions of exposure to known social determinants of health, and to maximise individual and community potential throughout all stages of life course (Marmot et al., 2010). By reducing social disparities in early developmental opportunities, standards of living, employment, and health care (Marmot et al., 2010), investment in these seven areas has been proposed to help reduce health inequities.

In 2015, a study by Frank et al. assessed how Scotland versus the rest of the United Kingdom (UK) ‘stacked up’ in terms of their implementation of these recommendations, as indicated by their respective national trends in health and socioeconomic outcomes (Frank et al., 2015). In recent history, Scotland had seen consistently higher levels of infant mortality (Palmer, 2010) and lower life expectancy (Kyte & Gordon, 2009) than the rest of the UK. In their study, Frank et al. found that Scotland had seen slightly greater reductions in child poverty compared to Wales and England in recent years, but lagged in achieving greater equity in relation to teenage pregnancy, early childhood education, educational attainment, employment, healthcare access, consumption of harmful food and drink, and gambling (Frank et al., 2015). A similar analysis has not yet been conducted for Canada.

Canada—like the UK—is considered a “liberal” welfare state (Esping-Andersen, 1990). Its delivery of social services draws from a protestant liberal tradition, and is marked by both high universal social insurance coverage (i.e. for sickness, unemployment, etc.) and high benefit differentials (i.e. benefits that are distributed unevenly in the population) (Van der Veen & Van der Brug, 2013). Canada’s universalist tradition is aligned in both theory and practice with values of equality and justice (Romanow, 2002), both of which underpin the Social Determinants of Health framework (Wilkinson & Marmot, 2003). However, since the 1970s, the country has been exposed to the policy paradigm of neoliberalism (Siddiqi, Kawachi, Keating, & Hertzman, 2013)—observed most recently through several periods of fiscally conservative leadership. Between 2006 and 2015, spending cutbacks occurred in housing, education, and social assistance programs—all of which are essential policy areas for the improvement of social determinants of health and health equity (Ruckert, 2012). Given the variability of political and moral frameworks that have guided policy and legislation in Canada over recent decades, it is useful to look at trends in equity outcomes across the seven areas of investment identified. The aim of this paper is to produce a ‘report card’ on Canada’s success in reducing inequities pertaining to the seven policy domains listed above, and wherever possible, to compare Canadian trends to those in the UK in order to benchmark Canada’s achievements in health equity against those in another liberal welfare state—one for which previous equity trend analyses have been performed. Identifying areas where Canada lags may help inform future research, policy and/or investments in the country. Further, differences between the two nations can highlight future areas for cross-national analysis of health and social policies, contexts, and interventions, and their differential impacts on health equity (Gilson, 2012).

## 2 Approach

This article summarises evidence from published studies, national reports and publicly-available summary statistics on health inequities in Canada and their determinants, and where possible, contrasts these trends with those observed in the UK. Data were identified through searches of Statistics Canada, Canadian Institute for Health Information (CIHI), Office for National Statistics (ONS), UK Government, and Organisation for Economic Co-operation and Development (OECD) web-based databases, as well as PubMed (for summary trend statistics in peer-reviewed publications). Snowball searches based on the reference lists of relevant peer-reviewed and grey-literature publications were also conducted to fill data gaps.

Instead of aiming to quantify Canada’s monetary investments in the seven areas identified (which can be very challenging when systems of national accounts vary across countries, as in this case), we focus on measurable outcomes related to socioeconomic inequities in these seven areas. To produce a summary ‘report card’ of trends in health equity-related outcomes in Canada the UK, we aim to summarize two features: the size of the change in the inequity through time (“Equity trend”) and the size of the remaining inequity at the latest data point (“Equity burden size”). Equity trend scores ranged from “Poor” to “Excellent” depending if the inequality increased, stayed stable, or decreased through time, whereas equity burden size scores ranged from “Poor” to “Excellent” if large versus very small/unsubstantial inequities remained. An average of these two scores was estimated. If the country’s two individual scores were consecutive in ordering (e.g. “Good” and “Excellent”) the lowest of two scores was up-weighted for more conservative estimation of “average” scores (i.e. the average between “Good” and “Excellent” scores would be “Good”). Used primarily to facilitate knowledge synthesis, the precision of these scores should be interpreted cautiously.

As with previous work (Frank et al., 2015), this study argues that socioeconomic inequities in these seven outcome categories are likely to be reduced following appropriate equity-oriented policy and program investments. We interpret trends in socioeconomic inequities in the seven areas as makers of potential success or failure of investments made. Focusing on trends at a national level in Canada, rather than at a provincial level, allows us to both capture how the sum of investments across provincial and federal jurisdictions influences average national outcomes, and to compare Canadian findings with those of other countries.

## 3 Equity trends: Seven key investments to improve health equity

### 3.1 Sexual and reproductive health, family planning, and pre- and perinatal care

Sexual and reproductive health, family planning, and pre- and peri-natal care are grouped here given their common ties to gender empowerment, and to intra-uterine, infant and child development. Family planning services are associated with fewer unintended pregnancies, and positive effects for the health and survival of the birthing individual (a term used here to be inclusive of transgender and non-binary individuals designated female at birth (Goldberg, Harbin, & Campbell, 2011)) and the child, as well as household poverty alleviation (Singh, Darroch, Ashford, & Vlassoff, 2009). Pre- and peri-natal care are also associated with improvements in child survival and birthing individuals’ health (Bryce, Black, & Victora, 2013). In turn, fetal and early childhood development influence later-life outcomes—particularly cardiovascular, respiratory, and endocrine health outcomes (Wilkinson & Marmot, 2003). Socioeconomic inequities in early life therefore tend to translate into inequities in health throughout the life-course (Kuh, Ben-Shlomo, Lynch, Hallqvist, & Power, 2003).

**Fig. 1:**
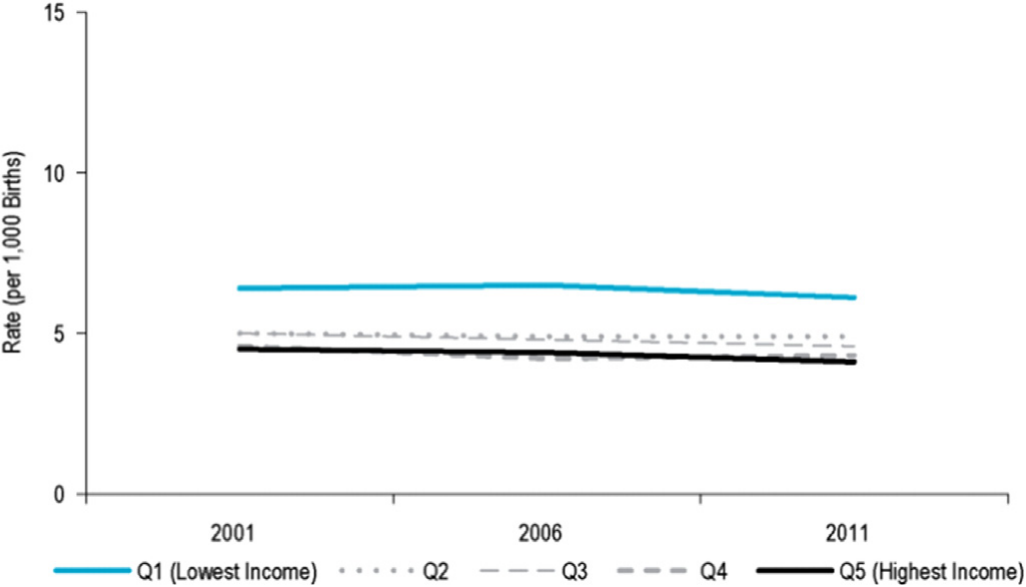
Infant mortality rates, by **area-level income** quintile, Canada (2001–2011). Image source: CIHI, 2016. Trends in Income-Related Health Inequalities in Canada: Technical Report.

**Fig. 2:**
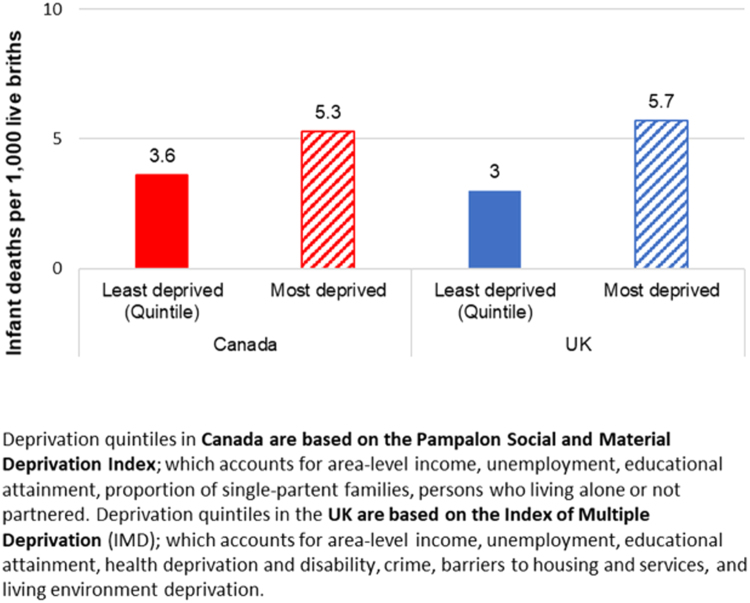
Average infant mortality rates between 2008 and 2011 according to local **area-level deprivation** in England (based on Index of Multiple Deprivation [IMD] scores) and in Canada (based on Pampalon Social and Material Deprivation Index scores). Graph created using data from: ONS (2016) Births and infant deaths in England, ref. 005621; and PHAC (2018) Canadian data from the Key Health Inequalities in Canada: A National Portrait [Annex 1].

Equity trends in reproductive health and care can be assessed through several proxies. Here we focus on infant mortality. Despite large decreases in infant mortality overall and across income groups between 1971 and 2001 in Canada (PHAC, 2008, Wilkins, 2007), rates have plateaued since and absolute income-based inequalities in infant mortality remain stable, but very small (Fig. 1). (CIHI, 2016b). When considering inequalities according to area-level social and material deprivation, there were on average 5.3 infant deaths per 1000 live births in the most deprived areas compared to 3.6 deaths/1000 in the least deprived areas between 2008 and 2011 (PHAC, 2018) (Fig. 2). No extant studies, reports, or statistics from the UK offered comparable data on trends in infant mortality according to area-level income, specifically. However, available data on infant mortality according to area-level deprivation between 2008 and 2011 suggest that the UK also observed a small remaining inequality between most- and least-deprived areas (ONS, 2016a) (Fig. 2). Though the UK’s area-level Index of Multiple Deprivation (IMD) uses a much wider range of factors compared to Canada’s area-level Pampalon Deprivation Index (ONS, 2016b) (Fig. 2), components of education, income and employment—three of the Pampalon Index’s six components—are given the largest weights (68.5%) in IMD score estimation (Kontopantelis et al., 2017). We therefore interpret the inequalities in Fig. 2 as broadly comparable.

### 3.2 Labour market and tax policies to reduce child poverty

Healthy child development relies on access to adequate physical, intellectual, and emotional resources around the child (Marmot et al., 2010). Poverty in these early years reduces the resources available to children and caregivers (Marmot et al., 2010), shapes household relations and stressors (Evans & Kim, 2013), and affects early educational outcomes (Bradbury, Corak, Waldfogel, & Washbrook, 2015). As developmental trajectories in the early years tend to determine social and health outcomes throughout the life-course (Wilkinson et al., 2003), child poverty reduction is identified as a top priority for reducing health inequities throughout the life-course (Marmot et al., 2010).

**Fig. 3:**
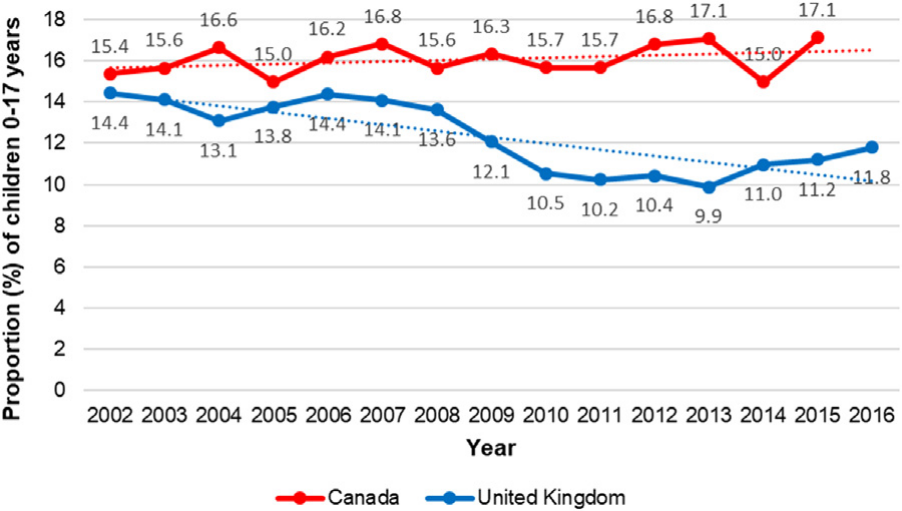
Percentage of population aged 0–17 years living in households with less than 50% of median household income between 2002 and 2016, in Canada and the United Kingdom. Graph created based on C02.2 Child Poverty OECD data (OECD 2018).

Equity trends in this area of investment can be assessed through trends in child poverty. The OECD provides data on child poverty, where poverty is measured using the cut off-value of 50% of median household income, adjusted for household composition (OECD, 2018b). Between 2002 and 2015, small increases in child poverty were observed in Canada—with 17% of children under 17 years living in poverty in 2015, an arguably large proportion of the population (Fig. 3). In contrast, in the United Kingdom, a small reduction in child poverty was observed during the same period, with a moderate prevalence of child poverty remaining in 2016 (12%) (Fig. 3) (OECD, 2018b). A limitation of these statistics for both countries, however, is that household income does not capture the availability or affordability of resources, or housing costs (Unicef, 2012). Lone parent families, for example, tend to be disproportionately burdened by both poverty (ONS, 2016c, StatisticsCanada, 2017) and housing costs (ONS, 2016c).

### 3.3 Early learning and child care

Early childhood education and child care (ECEC) plays a key role in equitable child development, and can greatly mitigate the life-course effects of adverse family and societal functioning of at-risk children (Loeb, Bridges, Bassok, Fuller, & Rumberger, 2007). Universal, high-quality pre-school for children aged two to five years tends to foster positive behavioral and cognitive development and school-based performance (Loeb et al., 2007), and is especially protective for children in low-income households (Dearing, McCartney, & Taylor, 2009). Beyond its positive impacts on child development, ECEC enables parents to work (McCuaig & Akbari, 2018)—thereby offering opportunities both for gender-based labour equality (and financial independence) and for increased household income (Heckman, 2011). In settings with limited public ECEC availability, parents either take on care work themselves, rely on family members, or purchase services—at potentially high opportunity costs for lower income households. Inequitable access to child care affects the purchasing power of household (influencing the availability of other resources and services in the home (Himmelweit, 2007)) and carves early economic inequalities in school-based success (Dearing et al., 2009)—thereby influencing education-based disparities in health throughout the life-course.

**Fig. 4:**
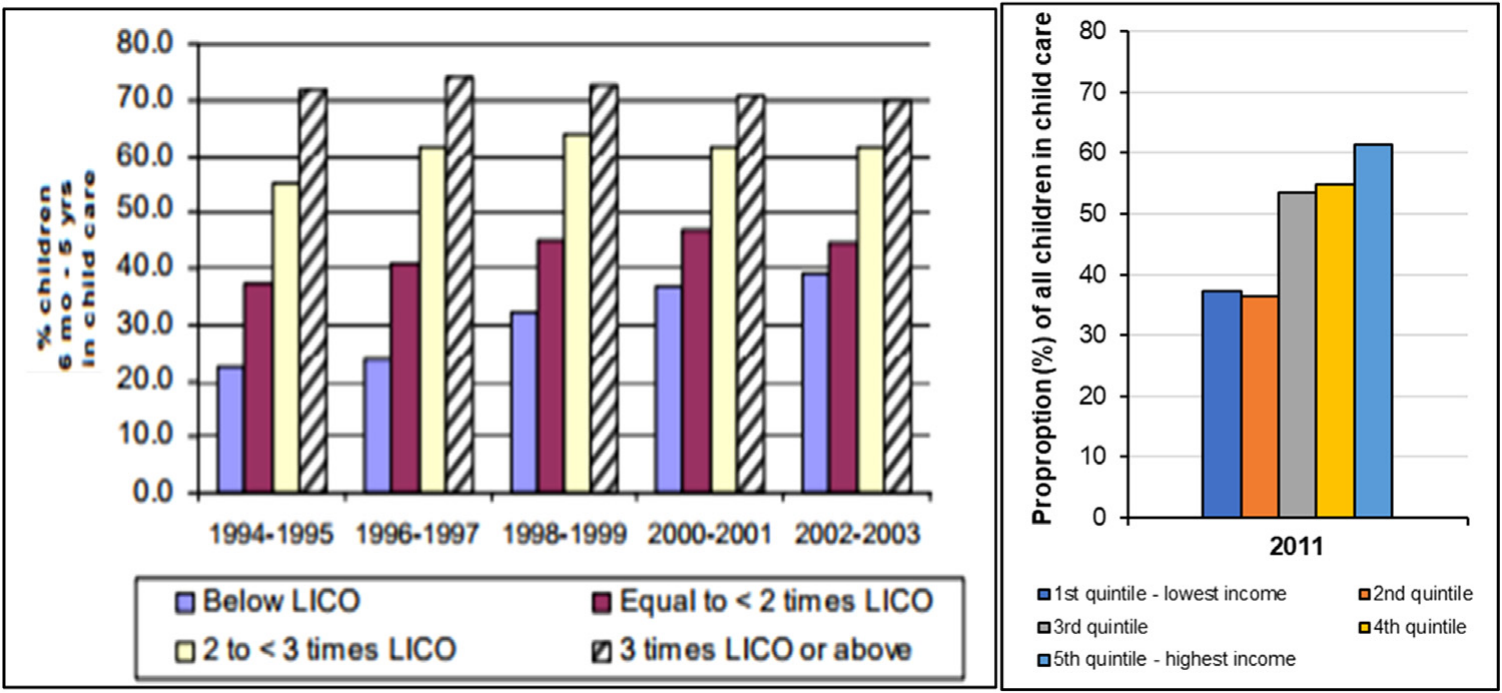
(Panel 1) The proportion of children aged 6 months to 5 years in non-parental child care by level of **household income** in Canada (defined according to the Low-Income Cut-Off (LICO) for each year (1994–2003). Image source: Bushnik, 2006; (Panel 2) The proportion of all children using child care according to household income quintiles in Canada (2011). Data source: Statistics Canada, 2011 (General social survey Cycle 25 – Family, 2011 accessed via ODESI Scholars Portal).

Equity trends in this area of investment can be assessed through trends in socioeconomic inequalities in child care use. Between the mid-1990s and early 2000s in Canada, the proportion of children aged 6 months to 5 years receiving child care increased, and a small decrease in the income-based inequality in child care use was observed (Fig. 4, Panel 1) (Bushnik, 2006). Despite these improvements, a large household income-based gap in early child care use persists in Canada (Fig. 4, Panel 2) (StatisticsCanada, 2011). Comparable UK trend data on children’s enrolment in child care or early childhood education programs according to parental income were not available for cross-national comparisons. UK data document trends according to area-level deprivation instead (Huskinson et al., 2017, Smith et al., 2009).

To be able to benchmark Canadian trends in this area of investment against those in the UK, two alternative indicators were used. First, since children who participate in more than one year of pre-primary education report much higher standardised learning scores (equivalent to over a year’s worth of schooling) than their peers who did not attend any pre-primary education (OECD, 2014a), we compared countries’ trends in standardised science scores (which measure scientific literacy among 15-year-olds (OECD, 2014b)) according to families’ socioeconomic status, measured using PISA’s index of economic, social and cultural status (OECD, 2016c). Second, since single parents tend to report lower income and purchasing power than two-parent households (ONS, 2016c, StatisticsCanada, 2017) and can therefore face challenges in accessing employment-enabling child care services, we assessed trends in employment rates among mothers with one or more dependent children aged younger than 14 years according to partnership status. The latter comparisons were performed within educational attainment sub-groups to account for potential associations between education and employment.

**Fig. 5:**
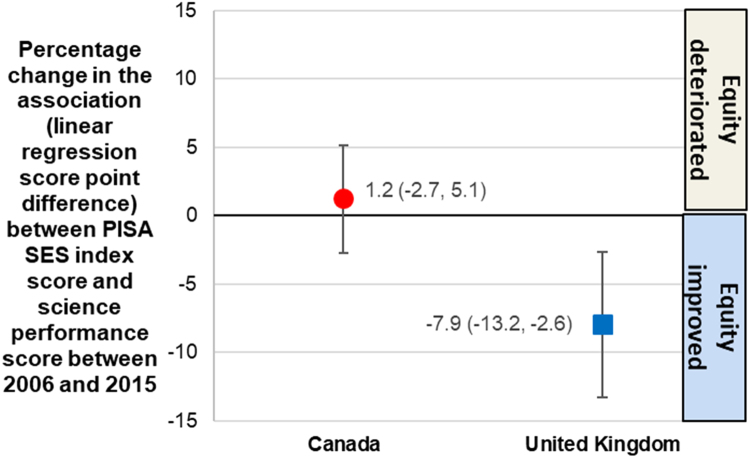
Change in the socio-economic gradient of performance scores for Canada and the United Kingdom between 2006 and 2015, measured as the change in the association (linear regression score point difference) between PISA’s index of economic, social and cultural status (ESCS) scores and science performance scores in both years. Graph created using data from Table I.6.17, OECD, 2016b.

**Fig. 6:**
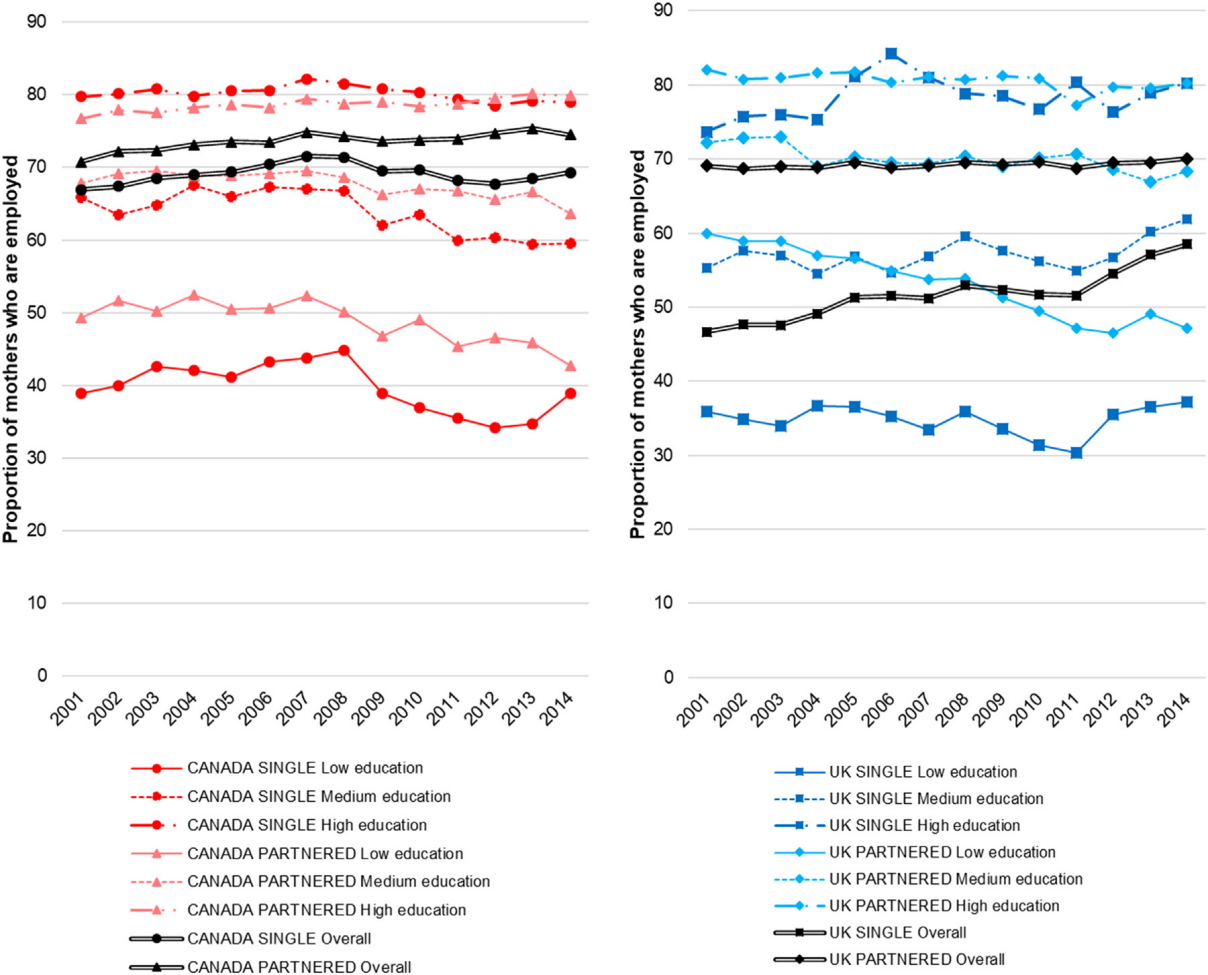
Employment rate among mothers of at least one child aged 0 to 14 years, in Canada (Panel 1) and the United Kingdom (Panel 2), between 2001 and 2014, according to mothers’ marital status and educational attainment (‘Low’ for pre-primary, primary or lower secondary education; ‘Medium’ for upper secondary and post-secondary non-tertiary education; ‘High’ for tertiary education. Graph created using OECD labour market position of families (LMF) Table 1.3 data from (OECD, 2018b).

Between 2006 and 2015 in Canada, science performance scores were stable and no change in the overall association between family socioeconomic status and science performance was observed (95% confidence intervals in the difference between the two years crossed the null) (Fig. 5) (OECD, 2016b). Overall in 2015, a moderate inequality in science scores existed between children of the lowest and highest quarters socio-economic status (OECD, 2016b). In contrast, the UK saw a reduction in the association between family socioeconomic status and science performance scores (indicating a reduction of the inequality) (OECD, 2016b), with a moderate inequality remaining in 2015 (Fig. 5). With regards to maternal employment, inequalities between single and partnered mothers in each educational attainment sub-group were stable through time in Canada (Fig. 6, Panel 1) (OECD, 2018b). Where employment levels dropped (especially after 2008), decreases appeared to affect both single and partnered mothers equally. In contrast, larger reductions in the employment inequality were observed, especially among lower-education sub-groups (Fig. 6, Panel 2) (OECD, 2018b). Nonetheless, a larger employment inequality between single and partnered mothers remained in the UK in 2014 compared to Canada, especially in less educated groups.

Performance scores and maternal employment were used here as indicators of access to early childhood learning and child care. However, both indicators have limitations. Performance scores are measured in adolescence, which means exposures incurred between early childhood and performance evaluation could have influenced score differentials. Further, though maternal employment may be influenced by child care availability, mothers who have access to child care may chose not to work for a variety of reasons—thus introducing potential bias in using this measure as an indicator. If trend data on alternative indicators become available, they may be warranted to perform sensitivity analyses of the above findings.

### 3.4 Universal secondary and higher education

Educational attainment is considered a key social determinant of health (Marmot et al., 2010). It is a marker of social status (or of status potential) within a society, and is tied to concurrent, everyday life exposures and experiences that influence mental and physical health outcomes, including income level, job security, work conditions, social networks, learned behaviours, and lifestyles (Backlund, Sorlie, & Johnson, 1999). Necessary for population-level educational attainment—particularly equitable distributions of educational attainment—are educational services marked by features of accessibility, affordability, and quality. Without these features, socioeconomic gradients in educational attainment and qualification are both produced, and perpetuated inter-generationally (Haveman & Smeeding, 2006).

**Fig. 7:**
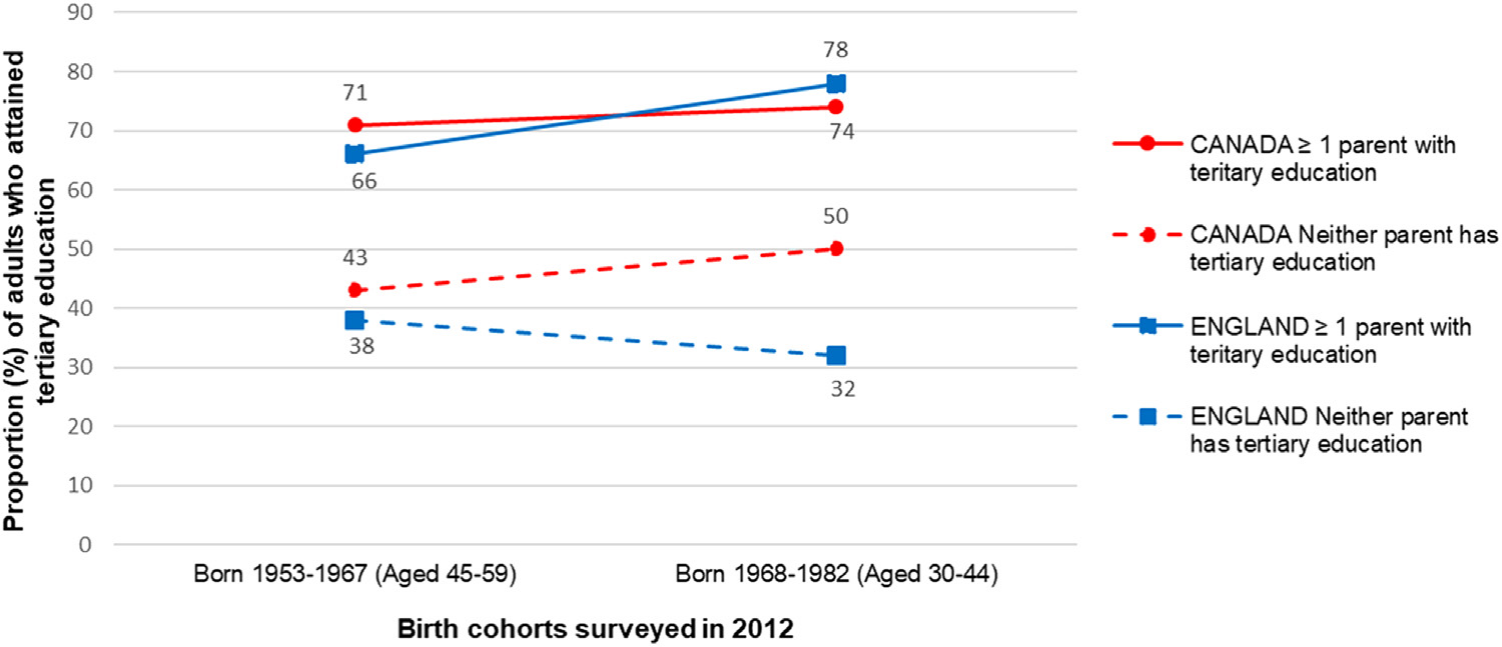
Tertiary education attainment in Canada and England in 2012 among two birth cohorts, adults aged 30 to 44 years (born between 1968 and 1982) and adults aged 45 to 59 years born (born between 1953–1967) according to parental tertiary attainment (neither parent has attained a tertiary level of education; or one or more parent has achieved tertiary education). Graph created using data from OECD 2018a, accessed via the OCEDstat database' section on Education and Training - Intergenerational Mobility in Education).

Equity trends in educational access and utilisation can be assessed through several proxies—including socioeconomic gradients in educational attainment—specifically for higher education. The OECD provides data on tertiary educational attainment (bachelor’s degree and above) in Canada and the UK, according to parental educational attainment (OECD, 2018a)—a marker both of socioeconomic equity in higher education attainment and intergenerational mobility. Among adults surveyed in 2012, a small decrease in the inequality in tertiary attainment according to parental educational attainment was observed between cohorts born before and after 1968 (i.e. aged 45 to 59 years versus aged 30 to 44 years) in Canada (Fig. 7). In the younger cohort, the gap in attainment between those with and without a parent who attained tertiary education remains large (Fig. 7). In England, a large increase in the inequality in tertiary attainment according to parental educational attainment was observed between the two cohorts; with a large inequality present in the younger cohort in 2012 (Fig. 7).

### 3.5 Accessible and high-quality primary, secondary, and tertiary care

High-quality universal health care is defined as promotive, preventive, curative, and rehabilitative health coverage available to all, regardless of wealth or status (WHO, 2014). Universal care generally relies on a financing system that can ensure service affordability, so that those receiving care do not suffer financial hardship due to fees pertaining to care, and so that those who require care receive it—regardless of their ability to pay for it (WHO, 2014). By preventing impoverishment due to out-of-pocket healthcare costs, which often produces greater socioeconomic divides, universal healthcare is considered an important strategy for poverty reduction and for the reduction of social inequities (WHO, 2014). It is also an important determinant of population health across the life course and societal productivity (WHO, 2014).

Canada’s publicly funded universal health insurance plan currently does not guarantee insurance for prescription drugs, longer-term care services, dental care, corrective lenses, or psychological care (Naylor, 1999). Public coverage for these services varies across provinces, and most Canadians have purchased private insurance to cover these extra costs (Naylor, 1999). Private prescription drug insurance—which is obtained by 60% of Canadians through their employer—pays for approximately 35% of drug expenditures in the country (Kratzer, Cheng, Allin, & Law, 2015). Those without private insurance pay for costs out-of-pocket. In contrast, private insurance plays a smaller role in the UK (i.e. representing only 4% of health expenditures) (Blendon et al., 2002). The UK’s National Health Service provides more generous subsidies for dental services and drug costs (Naylor, 1999)—especially in Scotland, Wales, and Northern Ireland where prescription charges are covered. Further, across the UK, certain populations (children, those on welfare, and with certain medical conditions) are entitled to free dental treatment (Bevan et al., 2014).

**Fig. 8:**
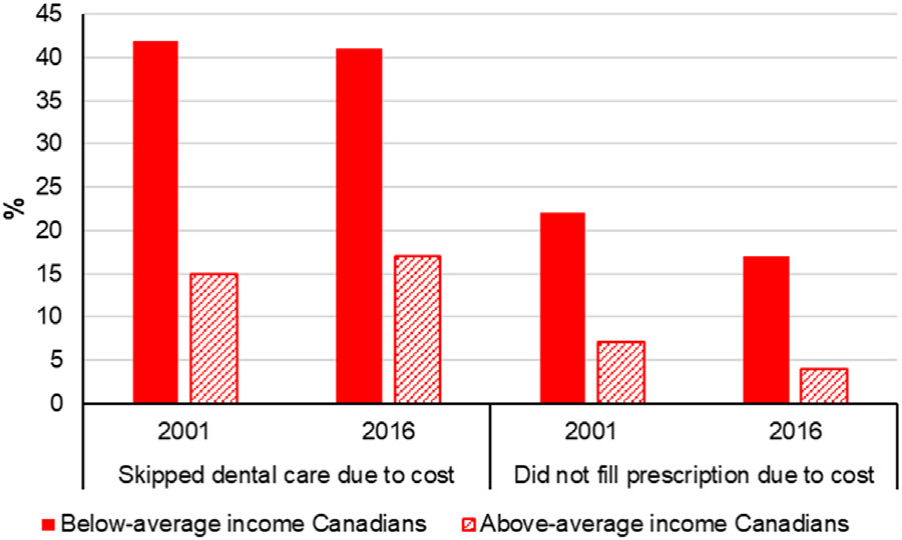
Proportion of Canadian respondents having not filled a prescription due to cost or having skipped dental care due to cost in 2001 and 2016, by income group. Graph created using data from Blendon (2002) and CIHI (2016a).

**Fig. 9:**
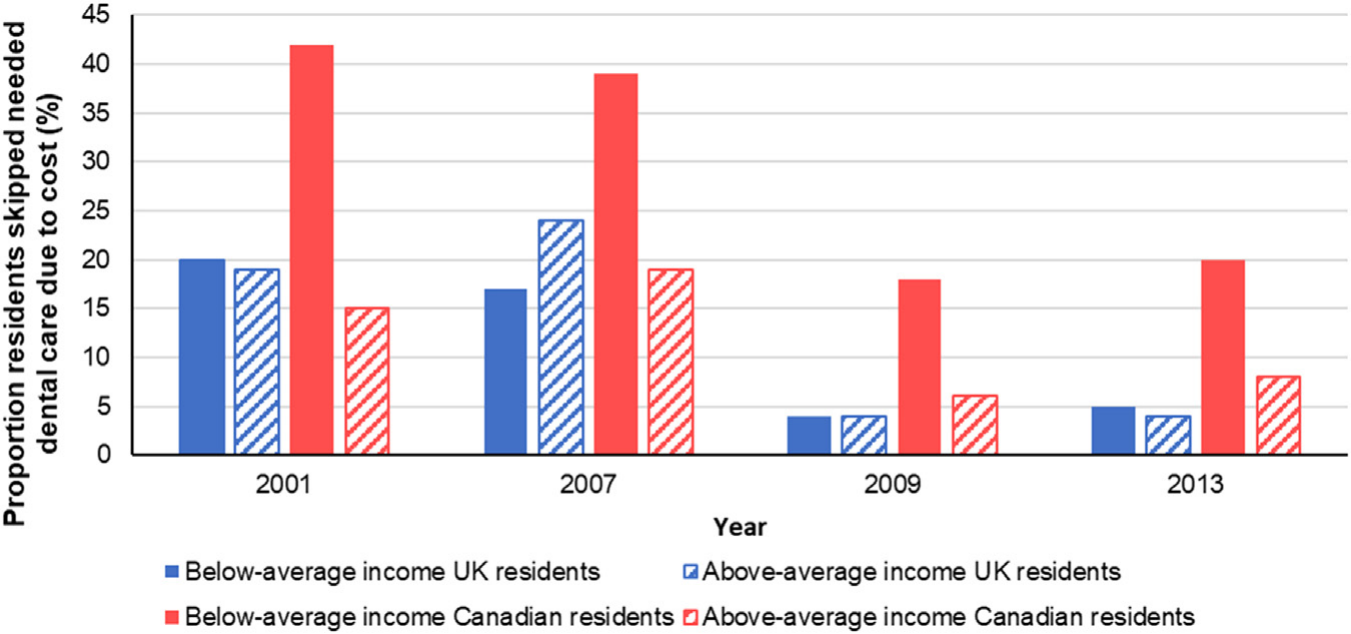
Proportion of residents of Canada and the United Kingdom reporting having skipped dental care due from 2001 to 2013, by income group. Graph created using data from Blendon (2002), OECD, 2009, OECD, 2011, OECD, 2015.

**Fig. 10:**
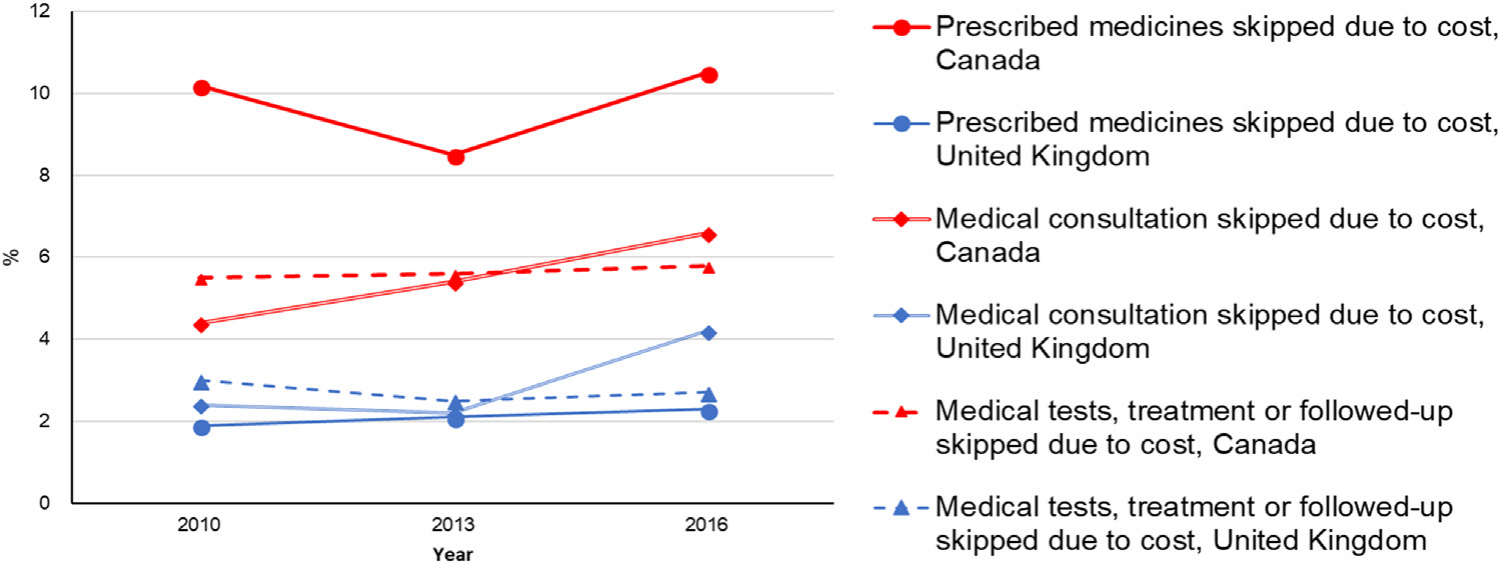
Proportion of residents of Canada and the United Kingdom reporting having skipped filling prescriptions, medical consultations, tests, treatment or follow-up due to costs from 2010 to 2016. Graph created using data from the OECDStat database (2016) section on Health - Health Care Quality Indicators.

Equity trends in accessible health care can be assessed through measures of reported economic barriers to care. In Canada, the proportion of individuals reporting barriers to care due to costs is highest among individuals with below-average income (CIHI, 2016a). Between 2001 and 2016, the proportion of low-income individuals who reported skipping dental care or prescription-filling due to costs dropped only slightly, while higher-income individuals’ saw a decrease in prescription skipping and an increase in dental care skipping (Fig. 8) (Blendon et al., 2002, CIHI, 2016a). Income-based inequities in access to dental care are larger in Canada than in the UK (Fig. 9) (OECD, 2009, OECD, 2011, OECD, 2015). In the UK, income-based disparities in dental care access grew between 2001 and 2007, but were virtually eliminated thereafter (Fig. 9)—likely, in part, due to dental care subsidies for low-income residents (Bevan et al., 2014). Overall, Canadians report greater cost-related barriers in filling prescriptions, and seeking medical consultations, tests, treatment or follow-up than UK residents, and the reported proportion of Canadians facing these barriers has not decreased since 2010—instead, some trends are on the rise (Fig. 10) (OECD, 2016a).

### 3.6 Economic and marketing controls of health hazards

Tobacco, excessive quantities of alcohol, ultra-processed foods (defined by their high energy density, glycaemic load, and fat, sugar and sodium content) (Moodie et al., 2013), and activities such as harmful gambling can be considered ‘health hazards’ insofar as they can negatively affect physical and psychological health (Korn, Gibbins, & Azmier, 2003). Each of these hazards tend to be marketed, for profit; and attempts to control their marketing are often resisted by vested interests (Frank et al., 2015). The availability and marketing of these hazards tend to target populations of lower socioeconomic status (Lee et al., 2015, Smoyer-Tomic et al., 2008, Wardle et al., 2014), placing these groups at higher risk of use and dependency. Indeed, economic gradients in gambling (Korn et al., 2003) and consumption of tobacco (Hiscock, Bauld, Amos, & Platt, 2012), high-dose alcohol (Schmidt, Mäkelä, Rehm, & Room, 2010), and ultra-processed foods (Miura, Giskes, & Turrell, 2012) place lower socioeconomic status groups at higher risk of facing social, health-related, and cost-related burdens of consumption. Several controls have been proposed to deter consumption these health hazards (Frank et al., 2015). These include interventions such as advertising and marketing bans, mechanisms to increase costs and restrict sales (Frank et al., 2015).

**Fig. 11:**
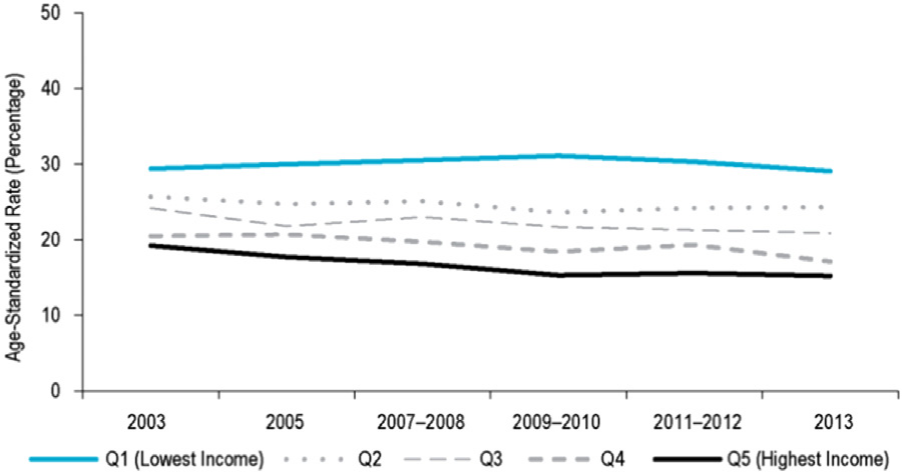
Age-standardised smoking prevalence rates, by **individual-level income quintile** in Canada between 2003 and 2013. Image source: CIHI Trends in Income-Related Health Inequalities in Canada, 2016.

**Fig. 12:**
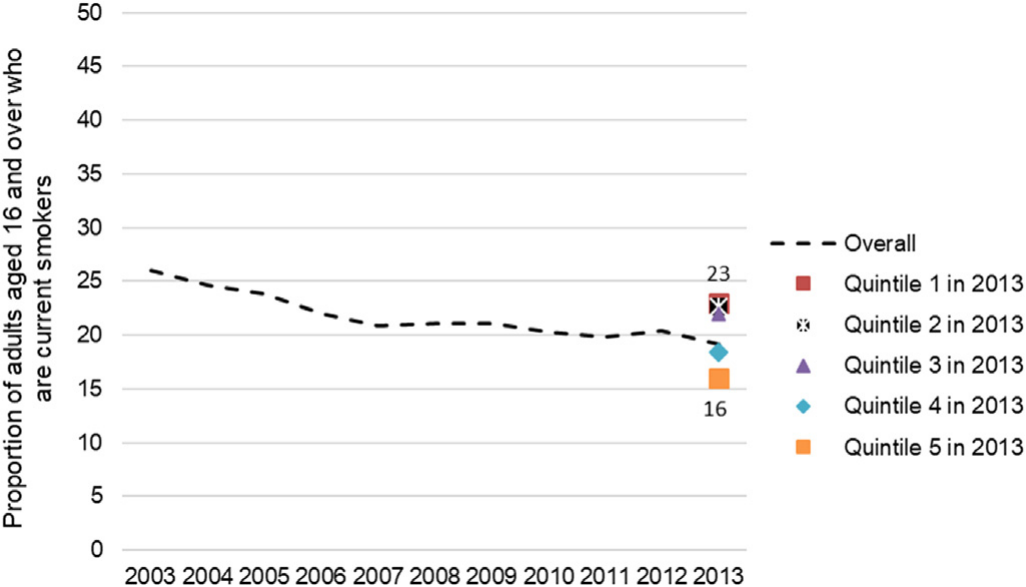
Cigarette smoking prevalence in England between 2003 and 2013 overall, and by individual-level income quintile in 2013 (only cross-sectionally data were available). Graph created using data from tables 6 and 14, Adult smoking habits in Great Britain 1974–2014 dataset (ONS, 2018).

Equity trends in the control of health hazards can be assessed through socioeconomic disparities in the consumption of several substances (Frank et al., 2015). Here we describe trends in tobacco use. Beginning in the late 1980s (Asbridge, 2004), several economic measures and marketing restrictions were implemented across Canadian jurisdictions (Non-smokers’ Health Act. c. 15 (4th Supp.), 1985, Tobacco Act (c.13), 1997)) to restrict smoking in federal work places, tobacco sponsorship and advertising, product displays, and to prohibit tobacco sale to minors. Now all provinces and territories have also implemented measures to restrict smoking in workplaces and public places (CCS, 2017). Indeed, regular smoking prevalence declined significantly in Canada since the 1980s (from 40% in 1980 to 24% in 2003) (HealthCanada, 2012), but the decline has tapered in recent years (i.e. from 24% in 2003 to 21% in 2013) (Fig. 11) (Minaker, Manske, Rynard, Reid, & Hammond, 2014). Largest decreases in smoking prevalence occurred among those with higher income—leading to small increases in income-based inequalities in tobacco use (Fig. 11) (CIHI, 2016b). As of 2013, a moderate income-based disparity in smoking prevalence remained in Canada (Fig. 11) (CIHI, 2016b). In the UK, where similar bans on smoking in public spaces, and restrictions of tobacco sale have been implemented (Frank et al., 2015), no extant studies or reports offer comparable trend data on smoking according to individual-level income (trends have mostly been measured according to area-level deprivation (Frank et al., 2015)). However, available cross-sectional data suggest that a moderate income-based inequality in smoking also remained in the UK in 2013 (Fig. 12) (ONS, 2018).

It should be noted that the latter trends were assessed before both countries introduced more aggressive tobacco control measures, such as legislation to ensure plain, standardized packaging of tobacco products, in 2016 (Norris & Tiedemann, 2016; The Standardised Packaging of Tobacco Products Regulations, 2015). Once data become available, more recent trends in social inequalities in smoking merit attention.

### 3.7 Sustainable economic development policies to support meaningful employment

A cyclical, inter-generational association exists between lower income, educational attainment, and under/unemployment (Heath et al., 2008, Lander et al., 2012). At a macroeconomic level, higher unemployment drives income inequality, thereby lowering aggregate demand and economic growth, and perpetuating higher unemployment (Dosi, Pereira, Roventini, & Virgillito, 2017). Operating through pathways of social isolation, lower self-esteem, and uptake of riskier behaviours (Bartley, 1994), unemployment is associated with negative mental and physical health outcomes, including higher psychological distress (Paul & Moser, 2009), chronic illness (Bartley & Plewis, 2002), lower self-rated health (Popham, Gray, & Bambra, 2012), and higher risk of mortality (Roelfs, Shor, Davidson, & Schwartz, 2011).

**Fig. 13:**
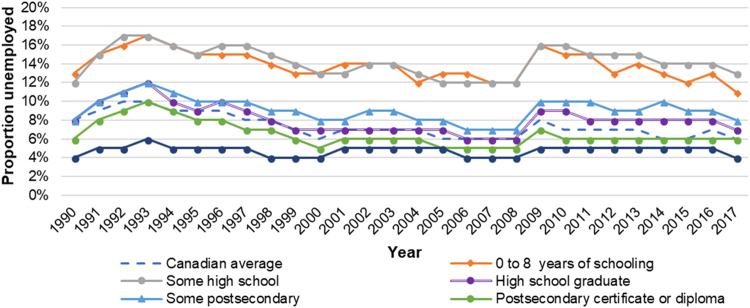
Unemployment rate by educational attainment among individuals aged 15 years and older in Canada, 1990–2017. Graph created using data from Statistics Canada CANSIM Table 282-0004 (Statistics Canada, 2018).

Equity trends in employment can be assessed through unemployment disparities according to individuals’ educational attainment. These disparities can be affected by policies (e.g. market stimuli, work incentives, job creation schemes), investments in education and vocational training (Bonoli, 2010, Frank et al., 2015), and by changes in national and global markets—most recent of which was the 2008 global economic recession. In Canada, the 2008 recession was associated with an increased prevalence of low-pay, precarious work, and an increase in unemployment (i.e. persons in the labour force who were without work, had looked for work in the past four weeks, or were waiting to start work in the next four weeks) (CIHI, 2016b). Those with lower education experienced largest rises of unemployment (Fig. 13) (CIHI, 2016b). In 2017, unemployment was 6% in Canadian adults aged 15 years and above, and the unemployment disparity between the lowest and highest education groups was approximately 7% (Fig. 13) (StatisticsCanada, 2018).

**Fig. 14:**
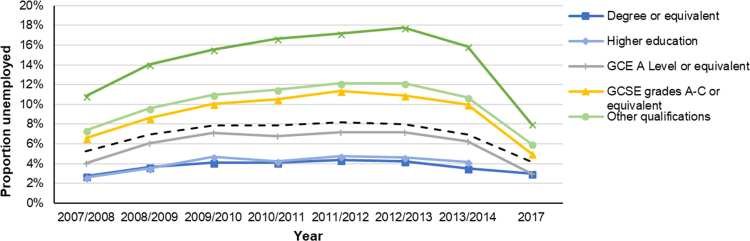
Unemployment rate by educational attainment for individuals aged 16–64 years in the United Kingdom (2007–2014) and (2017). Graph created using data from the UK Annual Population survey (Office for National Statistics (ONS), 2015), data from the Labour Force Survey (Office for National Statistics (ONS), 2017).

In the UK, unemployment captures the proportion of the labour force (aged 16 and above) without a job, who had looked for work in the past four weeks or were waiting to start within the next two weeks. Post-recession in the UK, the education-based disparity in unemployment among those 16 and above first increased, then decreased by 2017 (Fig. 14). Average unemployment was 4.2% in 2017, and an approximate absolute 5% gap in employment was observed between the lowest and highest education groups (ONS, 2015). It should be noted that these UK employment rates may not cover all economic migrants from the European Union (Gregg et al., 2010).

## 4 Discussion

The aim of this study was to assess national trends in health and socioeconomic outcomes in Canada according to seven broad areas of investment for health equity, and to assess how these trends compare to those in the UK, a fellow high-income “liberal” welfare state. Canada demonstrated heterogeneous trends in socioeconomic and health inequalities across the seven policy-investment domains studied (summarized in Table 1). First, its best successes were in improving equity in maternal employment and infant mortality. Improvements in the latter areas were largely equivalent to those in the UK in terms of both equity trends and the size of remaining inequities. Second, Canada saw only moderate improvements in equity in early learning outcomes, tertiary education attainment, barriers to health care, and employment. Canada’s equity trends in relation to early learning outcomes were similar to those observed in the UK. However, its success in improving equity in tertiary educational attainment appeared to be better than what was observed in the UK, where inequalities in educational achievement according to parental education stayed largely stable through time. In contrast, Canada’s moderate gains in accessible and affordable health care and employment were more modest than those observed in the UK, where inequalities in access to health care due to cost were much lower, and where educational disparities in employment decreased more substantially through time. Lastly, Canada’s least promising equity outcomes were in relation to exposure to “marketable” health hazards (specifically, tobacco) and child poverty. Canada’s moderate remaining gap in smoking prevalence according to individual-level income was similar to that observed in the UK. However, its upward trend in child poverty prevalence—a prevalence that was high to begin with—comes in stark contrast to reductions in child poverty observed in the UK.

**Table 1:** Summary report card of trends in social and health outcomes across socioeconomic groups in Canada and the UK, based on available data and socioeconomic indicators.

	Canada			United Kingdom			
Key investments and their indicator(s)	Equity trends^a^	Equity Burden size^b^	Average Score^c^	Equity trends^a^	Equity Burden size^b^	Average Score^c^	Country with highest average score
**1. Sexual and reproductive health, family planning**							
Infant mortality	Fair	Excellent	Good		Excellent		Tie^d^
							
**2. Policies to reduce child poverty**							
Child poverty	Poor	Poor	Poor	Good	Fair	Fair	UK
							
**3. Early learning and child care**							
Childcare access	Good	Poor	Fair				
Maternal employment	Fair	Excellent	Good	Excellent	Fair	Good	Tie
Science scores	Fair	Fair	Fair	Good	Fair	Fair	Tie
							
**4. Universal secondary and higher education**							
Tertiary attainment	Good	Poor	Fair	Poor	Poor	Poor	Canada
							
**5. Universal health care**							
Reported cost-barriers to care	Good	Fair	Fair	Good	Excellent	Good	UK
							
**6. Control of health hazards**							
Tobacco control	Poor	Fair	Poor		Fair		Tie^d^
							
**7. Economic development**							
Unemployment	Fair	Good	Fair	Good	Good	Good	UK

NOTE: Where cells are empty, strictly comparable across the two countries were not available, due to socioeconomic indicator/measure used. Scores are attributed according to available indicator (as discussed in figures or the text).

a Equity trend (based on findings discussed in figures or in the text, and available indicators) scores: Excellent=Large to very large reductions in inequality; Good=Small reductions in inequality; Fair=Stagnant or stable trends (i.e. not worsening); Poor= Small to large increases in inequalities.

b Equity burden size (based on findings discussed in figures or in the text, and available indicators) scores: Excellent = Very small/unsubstantial remaining inequality; Good = Small remaining inequality; Fair = Moderate remaining inequality; Poor = Large to very large remaining inequality.

c “Average score” represents an average scoring of the equity burden size and equity trend size grades—with lowest of two consecutive scores (for Equity trend, Equity burden size) up-weighted for more conservative interpretations.

d “Country with highest average score” was based on available comparable Equity Burden Size data only.

Identifying areas where Canada lags may help inform future research, policy and/or investments in the country. With regards to child poverty, UNICEF’s Innocenti “league tables” indicate that reductions in child poverty can be achieved through investment in transfer and tax policies for families with children (Unicef, 2017). In Canada, households with children are entitled to both federal and provincial child and family benefits (amounts of which vary based on the household’s past-year income) (Overview of child and family benefits, 2017). In a 2002 study, the generosity of Canada’s benefit system ranked below the UK’s—namely because benefits were lower when housing and service costs were considered (Bradshaw & Finch, 2002). Future studies of investments and policies within Canada and between Canada and other nations may inform ways in which Canada can reach international benchmarks.

A limitation of the comparisons presented here, however, is our restriction to one or two indicators for each area of investment. Future work is warranted to assess how trends vary if other indicators are used. However, as we found in conducting this review, future endeavours may also be challenged by limited data availability. The OECD offers rich data on several health and social outcomes. However, OECD outcomes are often presented as national averages, rather than stratified across socioeconomic indicators such as individual or parental income or education, or area-level income or deprivation. Another limitation is that health equity surveillance reports from Canada and the UK often present cross-sectional findings rather than trends through time—the latter of which are essential to assess present successes or failures in the context of historical trends. Furthermore, the two countries tend to use distinct socioeconomic indicators to assess inequities, which limits the ability to make cross-national comparisons. For example, many sources of UK data measure inequities in relation individuals’ area-level deprivation whereas Canadian trends are often presented according to individual-level income or education, or area-level income. Each of these indicators captures potentially distinct exposures and social strata. For instance, certain low-income individuals may live in areas with health-promoting social and built environments. Similarly, high-income families who have higher purchasing power and status may live in areas that are deprived of protective health and social resources (and vice versa). Ideally, international comparisons would be made using consistent and comparable indicators to minimize the risk of exposure (and social strata) misclassification.

Where comparable data are missing, accurate portraits of the scope, magnitude, and trends in health inequities cannot be created—leaving gaps in evidence for future policy interventions. Ongoing developments in social and health data linkage in Canada and the UK represent exciting opportunities to track health equity trends across a diversity of outcomes. However, we call for international standards and official (e.g. WHO) guidance for comparable health and socioeconomic equity indicators. These comparative resources would enable cross-jurisdiction comparisons, and the identification of best practices for health equity promotion.

## Ethical statement

According to the University of Edinburgh’s Usher Institute of Population Health Sciences and Informatics Research Ethics Policy, this project was judged to pose no foreseeable ethical risks (level 1 risk), and was not required to undergo formal ethical review by the Usher Ethics Review Group.

## Conflicts of interest

The authors have no conflicts of interest to declare.

## Financial disclosure statement

This research did not receive any specific grant from funding agencies in the public, commercial, or not-for-profit sectors. AB is supported financially by a Canadian Institutes of Health Research (CIHR) Michael Smith Foreign Study Supplement and by a CIHR Vanier Canada Graduate Scholarship. JF is supported by, the core grant to Scottish Collaboration for Public Health Reserach and Policy (SCPHRP) from the United Kingdom's Medical Research Council (MRC), with half that support from the Scottish Chief Scientist Office (MRC grant number MR/K023209/1). The funders played no role in the conception, execution or reporting of the research.
